# Eusociality and Senescence: Neuroprotection and Physiological Resilience to Aging in Insect and Mammalian Systems

**DOI:** 10.3389/fcell.2021.673172

**Published:** 2021-06-15

**Authors:** Ysabel Milton Giraldo, Mario L. Muscedere, James F. A. Traniello

**Affiliations:** ^1^Department of Entomology, University of California, Riverside, Riverside, CA, United States; ^2^Graduate Neuroscience Program, University of California, Riverside, Riverside, CA, United States; ^3^Department of Biology, Boston University, Boston, MA, United States; ^4^Undergraduate Program in Neuroscience, Boston University, Boston, MA, United States; ^5^Graduate Program in Neuroscience, Boston University, Boston, MA, United States

**Keywords:** hymenoptera, termite, naked mole rat, lifespan, antioxidant, neurodegeneration, metabolism, polyethism

## Abstract

Are eusociality and extraordinary aging polyphenisms evolutionarily coupled? The remarkable disparity in longevity between social insect queens and sterile workers—decades vs. months, respectively—has long been recognized. In mammals, the lifespan of eusocial naked mole rats is extremely long—roughly 10 times greater than that of mice. Is this robustness to senescence associated with social evolution and shared mechanisms of developmental timing, neuroprotection, antioxidant defenses, and neurophysiology? Focusing on brain senescence, we examine correlates and consequences of aging across two divergent eusocial clades and how they differ from solitary taxa. Chronological age and physiological indicators of neural deterioration, including DNA damage or cell death, appear to be decoupled in eusocial insects. In some species, brain cell death does not increase with worker age and DNA damage occurs at similar rates between queens and workers. In comparison, naked mole rats exhibit characteristics of neonatal mice such as protracted development that may offer protection from aging and environmental stressors. Antioxidant defenses appear to be regulated differently across taxa, suggesting independent adaptations to life history and environment. Eusocial insects and naked mole rats appear to have evolved different mechanisms that lead to similar senescence-resistant phenotypes. Careful selection of comparison taxa and further exploration of the role of metabolism in aging can reveal mechanisms that preserve brain functionality and physiological resilience in eusocial species.

## Introduction

Eusocial animals, characterized by reproductive division of labor, cooperative brood care, and overlap of generations, can have extraordinary lifespans. Eusocial hymenopteran (ants, bees, and wasps) queens may live 100 times longer than solitary insects. In some eusocial species there is a similar lifespan differential between queens and workers (Keller and Genoud, 1997; Kramer and Schaible, 2013). Naked mole-rats (NMRs), exemplar eusocial mammals, also have highly extended lifespans compared to solitary rodents (Buffenstein, 2008) and do not exhibit typical age-associated increases in mortality (Ruby et al., 2018). These clades present opportunities to examine molecular and physiological processes underlying differential longevity, their degree of conservation, and relationships to social evolution (Lucas and Keller, 2020). Aging resilience is often manifest as a lack of deterioration in neural, reproductive, or immune function (Buffenstein, 2008; Finch, 2009; Stenvinkel and Shiels, 2019). Robustness to senescence in eusocial taxa may be associated with adaptations involving damage-repair mechanisms, neuronal protection, neurometabolic efficiency, and fossorial ecology, among other factors. Social development—age-related changes in behavioral role or task performance—may also be involved. Here we focus on senescence in eusocial insects and NMRs to identify commonalities in aging phenotypes, examine anti-aging mechanisms, and suggest future research.

## Caste Determination and Aging

Reproductive caste is determined nutritionally in honeybees, by nutrition and social factors in many wasps (O’Donnell, 1998; Berens et al., 2015), and by social, environmental, and genetic factors in ants (Schwander et al., 2010). In most cases, caste is determined early in development and remains fixed for life, although in a few taxa, trajectories are more plastic. In the ant *Harpagnathos salatator*, workers can facultatively become reproductive upon queen loss (Liebig et al., 2000; Peeters et al., 2000). Similarly, queens are replaced after dominance contests in NMRs (Clarke and Faulkes, 1997). Cape honeybee workers may become egg-laying pseudoqueens and increase their lifespan to 5 months (Rueppell et al., 2016). Therefore, social insects have plastic developmental trajectories of senescence. Changes in gene expression (Gospocic et al., 2017; Shields et al., 2018) can drive differentiation toward reproductive competence to influence lifespan. Reproductive status and longer lifespans correlate with a reduction in physiological aging and limited senescence in the brain (Finch, 2009; Parker, 2010; Giraldo and Traniello, 2014; Giraldo et al., 2016).

NMRs, which differ from mice in many physiological and biochemical markers of aging (Buffenstein, 2008), have a 30+ year lifespan and significantly delayed senescence (Lee et al., 2020). Although early studies reported lifespans between queens and subordinates were similar (Sherman and Jarvis, 2002), a more recent and comprehensive study revealed that NMR breeders, like many eusocial insects, have lower age-associated mortality than non-breeders (Ruby et al., 2018). Similarly, reproductives of the eusocial Damaraland mole rat are longer lived than non-breeders (Schmidt et al., 2013), suggesting convergence of aging phenotypes. However, longevity may not correlate with eusociality *per se* (Lucas and Keller, 2020) and selection can differ among species. The subterranean life of NMRs has been suggested to drive long lifespan (Healy et al., 2014), although it appears to have no effect across mammals after accounting for sociality (Healy, 2015).

## Developmental Timing and Neoteny

What does it mean to live a long time? Measuring lifespans simply as the interval between birth or hatching and death ignores significant variation across solitary and eusocial taxa in the timing and significance of developmental events related to senescence. Ant queen larval stages are relatively short and adults may live decades (Hölldobler and Wilson, 1990). Immature periodical cicadas and mayflies live for years, but adults live only days (Britain, 1990; Grant, 2005). Variation in lifespan and developmental timing is also evident across mammals (Healy, 2015; Healy et al., 2019). However, the relationship between developmental timing and aging in eusocial systems is unclear.

Eusociality may be associated with extended developmental periods and the retention of juvenile traits into adulthood (neoteny or pedomorphy; Orr et al., 2016) that may affect lifespan. Brain development in NMRs occurs at a significantly different pace than in mice. Although similar in size, NMRs are born with larger, more slowly developing brains. In eusocial insects, major developmental transitions occur from egg to adult and during age-related behavioral development within adulthood (Whitfield et al., 2003; Seid and Traniello, 2006). Eusocial insect neoteny could therefore be evident in delayed pupation or altered rates of adult maturation and behavioral development relative to solitary taxa. Indeed, ant species with shorter egg-to-eclosion development may have longer latencies to the onset of foraging, suggesting a tradeoff between development and behavioral maturation (Muscedere, 2011). Also, in the ant *Pheidole dentata*, newly eclosed minor workers possess undeveloped mandibular muscles (Muscedere et al., 2011) and small task repertoires lacking efficiency (Muscedere et al., 2009). Such morphological and behavioral immaturity would likely be costly in adult solitary insects. *P. dentata* minor worker brains collaterally undergo significant age-related changes in size, monoamine titer, and synaptic structure as they mature behaviorally (Seid and Traniello, 2005; Seid et al., 2005; Muscedere and Traniello, 2012; Muscedere et al., 2012, 2013). Whether worker “neoteny” is altriciality or accelerated larval and pupal maturation, the process appears to enable workers to eclose earlier as undeveloped adults. The influence of pace of development on aging and longevity remain unexplored.

The adaptive nature of developmental patterns and their distribution across eusocial species that vary in ecology and life histories is unstudied. Division of labor in NMR colonies among non-breeders can be influenced by age, with older individuals generally performing less work, but factors such as body size and rank can interact in non-linear ways (Gilbert et al., 2020). In NMRs, neoteny may be an adaptation to living in hypoxic burrows: neonatal mammal brains often have higher hypoxia tolerance than adult brains (Larson and Park, 2009). This could apply to some subterranean eusocial insects if their nests are hypoxic. Reductions in extrinsic mortality risk in eusocial animals arising from their well-defended nests could also select for neoteny, in addition to longer lifespans in general (c.f. Keller and Genoud, 1997), allowing relatively undeveloped individuals to safely labor (Muscedere, 2011).

## Neurobiological Resilience

Neural markers of social insect senescence suggest chronological age and neural deterioration, including DNA damage or cell death, are decoupled. Brain cell death does not increase with age in minor workers of the ant *Pheidole dentata* (Giraldo et al., 2016) and synaptic complexes in higher-order processing centers (mushroom bodies) do not change over up to 68% of their 140-day laboratory lifespan, suggesting a lack of neurodegeneration. Worker lifespan in the field is likely to be significantly shorter, rendering aging inconsequential in nature. *Drosophila melanogaster* exhibit apoptosis in muscle and adipose tissue, but do not show programmed brain cell death beyond neural remodeling post-eclosion (Zheng et al., 2005), although the antennal lobes and ellipsoid body, which are critical for olfaction and spatial orientation, respectively, exhibit elevated caspase-3-like activity (DEVDase) in older flies (Chihara et al., 2014). Antennal lobe apoptosis appears to be restricted to specific classes of olfactory receptor neurons causally related to age-related declines in olfaction (Chihara et al., 2014). In social insects, DNA damage occurs at similar rates between queens and workers (Lucas et al., 2017), but DNA repair gene expression is higher in the former (Lucas et al., 2016). Although honeybees exhibit generally low levels of oxidative damage in the brain, foragers show higher levels of protein carbonylation in the optic lobes than chronologically older overwintering bees (Seehuus et al., 2006a). Although chronological age and associative learning are not correlated in honeybees (Rueppell et al., 2007), increased foraging is generally associated with memory-associated declines (Behrends et al., 2007). Examination of brain compartment-specific changes in protein abundances suggest neural senescence may not be regulated at the level of the whole-brain (Wolschin et al., 2010). Together, these studies suggest that despite very different aging phenotypes, eusocial and solitary insects could share common mechanisms conferring aging resilience in neural tissue.

NMRs exhibit neuroprotective characteristics of neonatal mice such as protracted development (Buffenstein et al., 2020). Perhaps due to a focus on comparisons with mice, little research has explored whether queen and worker brains differ in neural markers of aging. In one study, transcriptomes of breeding NMRs were enriched for aging-related genes compared to their non-breeding littermates, although not in the brain regions measured (Bens et al., 2018). Unlike most mammals, few genes were found to be differentially expressed between 4 and 20-year-old NMRs, particularly in the brain (Kim et al., 2011), suggesting maintenance of aging-resistance throughout life. Alternative splicing, in which a single gene leads to different functional isoforms, has been suggested as an adaptive stress response and seems to be upregulated in NMR brains relative to mice (Lee et al., 2020). *In situ* comparison of NMRs and mice indicate that NMR neurons are more resistant to acid-induced cell-death (Husson and Smith, 2018).

Eusocial brains may also be robust to injury or disease. For example, unilateral antennectomy in newly eclosed *P. dentata* workers led to a significant reduction in ipsilateral antennal lobe volume but had few other neurobiological effects, and performance of most tasks remained unaffected, suggesting developmental neural resilience to damage at least early in adult life (Waxman et al., 2017). This is consistent with studies in solitary insects that demonstrated marked robustness to injury. Unilaterally antennectomized cockroaches are able to successfully track odor plumes (Lockey and Willis, 2015). By removing the distal segments of the antennae in hawkmoths during the beginning of the pupal stage, researchers removed sensory neurons, resulting in abnormal antennal lobe development; nevertheless, adults were able to successfully find the source of the odor plume in a wind tunnel (Willis et al., 1995). These findings suggest resilience in both solitary and social insects.

The NMR immune system appears well-adapted to combat bacterial infections (Hilton et al., 2019) but vulnerable to viruses (Artwohl et al., 2009), although the impact on general immunosurvellience has yet to be elucidated (Hilton et al., 2019). Immune-function genes appear to be upregulated in some bees and termites but not ants, suggesting a lack of universal pathways in social insect immune responses (Korb et al., 2021). Whole-body transcriptomic analysis of workers, queens, and kings of the termite *Reticulitermes speratus* show strong up-regulation of DNA repair genes in mature kings, indicating that DNA repair may be an important component of aging resilience (Tasaki et al., 2018). BRCA1, involved in DNA repair and antioxidant signaling among other functions, was highly expressed in mature kings. Mature queens and kings showed different levels of expression (Tasaki et al., 2018). This system, and hymenopterans, enable sex-related differences in reproductive longevity to be examined.

Glia may offer neuroprotection. Aging *D. melanogaster* and *H. saltator* workers show decreases in gene expression for transcripts that characterize ensheathing glia (Sheng et al., 2020), which in *Drosophila* respond to injury (Doherty et al., 2009; Kato et al., 2011, 2018) and decline in function with increasing age (Purice et al., 2016). However, age-matched *H. saltator* reproductives exhibit a higher proportion of these glia, suggesting a neuroprotective role. Old honey bee foragers exhibit lower levels of two glial metabolic enzymes, glutamine synthase and glycogen phophorylase, perhaps driven by declining protein expression and/or glial cell loss (Shah et al., 2018).

The association of eusociality and neurobiological resilience is unclear, although a general *lack* of resilience has historically been implied. Exposing workers to disease, injury, or carbon dioxide induces early foraging, as expected from manipulations that shorten lifespan and reduce worker residual value (Moroń et al., 2008; Tofilski, 2009). Old ant workers have been hypothesized to be “disposable” (Porter and Jorgensen, 1981), and in some species workers have been characterized as “cheap” and replaceable (Wilson, 2003). The costs and benefits of portioning risky tasks by age depends on aging rates and whether age-related mortality varies with task performance (Tofilski, 2002). Improved task performance by mature older workers may instead alter the colony-level fitness costs of worker maintenance or replacement, selecting for robustness, neural resilience, and extended lifespans. A rigorous test of these hypotheses would require controlling for phylogeny and body size.

## Antioxidants and Aging in Eusocial Species

Harman (1956) first hypothesized that reactive oxygen species (ROS) produced as byproducts of cellular metabolism result in accumulated molecular and cellular damage and ultimately degradation and death. This free radical theory of aging has been tested, with inconsistent results (Ashok and Ali, 1999; Ziegler et al., 2015; Grimm and Eckert, 2017). A key limitation of ROS theory in its most simplistic form is that it fails to explain the wide variation in lifespans across animals (Keller and Genoud, 1997; Rose et al., 2002). Nevertheless, as one of multiple potential aging mechanisms, experimental studies have examined ROS and antioxidant systems across taxa.

Insect fat body produces and processes hemolymph proteins and hormones implicated in aging (Amdam et al., 2004; Corona et al., 2007; Smedal et al., 2009). In *Drosophila*, expression of the antioxidant-related gene *catalase* (CAT) declines with age (Klichko et al., 2004), and overexpression of CAT and another antioxidant gene, *superoxide dismutase* (SOD), lowers levels of protein carbonylation (a measure of oxidative damage) and extends lifespan (Orr and Sohal, 1994). The pattern in eusocial insects, however, varies. Senescence-associated changes in brain and fat body gene expression in young and old queens in the ant *Temnothorax rugatulus* are highly tissue-specific (Negroni et al., 2019), but old queens exhibit higher levels of SOD and CAT. Queen termites, *Reticulitermes speratus*, also show lower levels of oxidative damage and CAT upregulation (Tasaki et al., 2017). CAT activity is higher in *R. speratus* compared to solitary insects and eusocial hymenopteran queens (Tasaki et al., 2017). In contrast, levels of most antioxidant genes in the brains of honey bee queens decline with age, whereas levels in worker brains remain constant or increase (Corona et al., 2005). Queen mRNA levels of these genes were often lower than in workers at least 1 week old, although this does not suggest honeybee queens lack pro-longevity repair mechanisms. Honey bee queens showed an upregulation of antioxidant activity in the fat bodies and trophocytes, accompanying an increase in ROS (Hsieh and Hsu, 2013). In contrast, ROS levels decline with age in worker honey bees, but antioxidant levels are constant or increase (Hsu and Hsieh, 2014). These studies suggest that ROS and antioxidant activity may involve tissue-specific regulation. Honeybee workers are protected from oxidative stress by the yolk precursor vitellogenin, an additional ROS protection mechanism (Seehuus et al., 2006b). Alternatively, *Lasius niger* queens invest in DNA and protein repair rather than antioxidants (Lucas et al., 2016). Oxidative damage can also be induced by stressful environmental conditions in honeybees (Simone-Finstrom et al., 2016), but does not necessarily result from lifespan reducing stress (Rueppell et al., 2017). In the reproductively plastic termite *Crypotermes secundus*, colonies maintained at constant temperature counterintuitively showed higher stress responses, lower survival, and reduced reproductive output than those at variable temperatures (Rau and Korb, 2021). Changes in gene expression were similar between queens and pseudergates (“false workers”), perhaps due to their reproductive plasticity (Rau and Korb, 2021). A comparative transcriptomic analysis of termite, bee, and ant species identified a few genes associated with increasing age and/or caste but no consistent patterns across taxa (Korb et al., 2021). ROS systems are complex, and manipulations of multiple systems will be necessary to uncover underlying genetic mechanisms of aging (De Verges and Nehring, 2016). Although some social insect queens exhibit higher levels of antioxidants, as expected given their long lifespans, this mechanism does not appear to be necessary in other species. Indeed, a recent comparative analysis of levels of oxidative damage and antioxidant genes in some ant, bee, and termite species found a marked lack of consistency (Kramer et al., 2021).

Like eusocial insects, the extreme longevity of the naked mole-rat does not completely align with the oxidative stress theory of aging (Lewis et al., 2013). ROS production—specifically in heart tissue—is similar to that observed in shorter-lived mice (Lambert et al., 2007; Munro et al., 2019), and equivocal findings have been reported for antioxidant defenses that are not only tissue and cell-site specific but also specific to different antioxidants (Andziak et al., 2005; Munro et al., 2019; Viltard et al., 2019; Takasugi et al., 2020). Notably, NMRs appear to sustain high levels of oxidative damage (Andziak et al., 2005, 2006; Pérez et al., 2009; De Waal et al., 2013; Lewis et al., 2013). However, NMRs exhibit higher levels of mitochondrial consumption of hydrogen peroxide in skeletal muscle and the heart than mice, suggesting improved ROS scavenging (Munro et al., 2019), and kidney protein function is maintained in NMRs despite high levels of protein carbonylation (De Waal et al., 2013). Levels of mitochondria-bound hexokinases, which can prevent ROS formation during cellular respiration, decline in many tissues including the brain in mice but are maintained for minimally a decade in NMRs (Vyssokikh et al., 2020). Membrane phosopholipids are more resistant to oxidation in NMRs than mice in many tissues, although their brains do not differ (Hulbert et al., 2006). These seemingly confusing findings suggests that the long lifespan of NMRs is not simply a result of less production or more scavenging of ROS. Instead, high levels of ROS observed early in life in long-lived vertebrates such as NMRs, birds, and bats could be adaptive, priming lifespan management of ROS (Saldmann et al., 2019). Direct comparisons with eusocial insects are hampered by methodological differences, but will be important to explore variation across taxa in ROS and antioxidant production as well as management of ROS-related damage.

## Age, Longevity, and Brain Metabolism

Eusocial insect worker and reproductive castes differ in metabolism, and metabolic pathways may correlate with aging phenotypes (Corona et al., 2007; Ihle et al., 2019; Haroon, Ma et al., 2020). Caloric restriction has a positive effect on lifespan and senescence in humans (Most et al., 2017), some genetic strains of mice (Weindruch, 1992; Liao et al., 2010) and *Drosophila* (Burger et al., 2010), suggesting that by lowering metabolic activity cells form fewer injurious metabolites (Speakman and Mitchell, 2011). Nevertheless, caloric restriction does not extend lifespan in all species (Speakman and Mitchell, 2011) and can even reduce longevity (Kaitala, 1991; Kirk, 2001; Liao et al., 2010). Resveratrol, which mimics the effects of caloric restriction, extended lifespan in worker honeybees while reducing food consumption (Rascón et al., 2012).

How does brain metabolism scale with longevity? Comparison of brain investment and energetic demands in ant species that differ in social complexity suggest tradeoffs between increased brain size and metabolism (Kamhi et al., 2016). A plastic shift in energetic investment appears to occur in the ant *Harpegnathos saltator*, in which gamergates that are experimentally reverted to foragers increase investment in their brains and decrease investment in the gonads, reversing patterns found in naturally occurring gamergates and workers (Penick et al., 2021). Reverted honey bee foragers did not exhibit brain shrinkage in the mushroom bodies after 5 days (Fahrbach et al., 2003), although the short time scale may not have been sufficient to see effects similar to *Harpegnathos* (Penick et al., 2021). These studies suggest that social insects may be able to modulate their brain volumes adaptively, although limits to this plasticity likely exist.

In honeybees and *Drosophila*, experimentally inhibiting oxidative phosphorylation pathways leads to increased aggression (Li-Byarlay et al., 2014). These effects can be socially modulated in honey bees (Li-Byarlay et al., 2014), potentially impacting aging and life history if it alters social roles. NMRs exhibit a point mutation in a neuronal potassium chloride cotransporter that lowers the energy costs of GABAergic signaling in low oxygen environments (Zions et al., 2020), intriguingly suggesting an interaction between brain metabolism and environment. Interactions between abiotic conditions and aging related genes in termites (Rau and Korb, 2021) suggest that metabolism-environment interactions could exist in many taxa. In old honey bee queens, oenocytes and trophocytes do not show declines in mitochondrial energy metabolism compared to young queens (Hsu and Lu, 2015). In contrast, workers experience age-related declines in energy-related molecules (Hsu and Chuang, 2014) and cellular metabolism (Lu et al., 2017).

## Future Explorations of Aging Among Eusocial Taxa

### Caste and Species Comparisons

Understanding age-related changes in neurobiology and behavior in long-lived ant queens requires knowing queen age. Fire ant queens (6–7 year lifespan) store sperm from a single insemination for life, allowing sperm counts to reliably estimate queen age (Tschinkel, 1987). Queens collected from established colonies can be aged and brains analyzed for neuroanatomical changes, synaptic structure, neurochemistry, and gene expression. By contrasting patterns between queens and workers, the influence of colony reproductive phenotype—monogyne or polygyne—can be determined to assess the role of social structure on aging. This single trait has been evolutionary labile in social insects and has multiple impacts on social phenotypes (Hölldobler and Wilson, 1977), including longer lifespans in monogynous than polygynous queens (Keller and Genoud, 1997).

Caste theory (Oster and Wilson, 1978; Wilson, 2003) can be applied to the neurobiology and physiology of aging, and the hypothesis that workers are “cheap or disposable” can be tested by estimating brain production and/or maintenance costs to understand the physiological underpinnings of minimal or discontinued investment. Additionally, neuroanatomical analyses in species capable of facultative switching between worker and reproductive castes can examine whether the transition to a long-lived reproductive activates neurobiological and physiological mechanisms that protect now reproductively competent individuals from senescence. Comparisons between taxa in which workers can assume a reproductive role—including termites and NMRs—will reveal whether independent evolution of reproductive plasticity involves common pro-longevity mechanisms.

Most aging comparisons with NMRs involve studies of other taxa. Mutant dwarf mice with growth hormone mutations exhibit pedomorphic traits and have at least 50% longer lifespans than wild-type mice, and fewer aging-associated diseases (Buffenstein et al., 2020). Experiments that present NMR non-breeders, breeders, and dwarf mice of different ages with environmental toxins or thermal stress could help separate the roles of sociality and reproductive status in aging.

### Metabolism and Aging

Brain metabolism is little explored in eusocial and solitary species (Neville et al., 2018; Coto and Traniello, 2021). Is energy use plastically regulated across caste and lifespan? Do queens and workers differ in brain metabolic scaling? Do subterranean social insects experience hypoxic or hypercapnic conditions and if so, have they evolved NMR-like neurometabolic adaptations to these environments? Queens of the termite *Reticulitermes speratus* reproduce in hypoxic, hypercapnic chambers, conditions that enhance their reproductive output (Tasaki et al., 2020), although the generality of this result is unclear. These questions should be comparatively addressed across eusocial and long-lived solitary species. Additionally, despite relatively long-lived NMR workers, gene expression differences between breeders and non-breeders exist in skin and gonads, tied to reproductive maturation and in some cases aging (Bens et al., 2018). Future studies could explore how reproduction and energy use affect brain metabolism that go beyond volumetric measurements in social insects. Although plastic changes in brain volume, as in *Harpegnathos saltator* (Penick et al., 2021) hint at metabolic tradeoffs, methods that can quantify brain metabolism (e.g., Neville et al., 2018) will allow researchers to directly test whether volume changes correlate directly with tissue energy consumption and how energy use may change across the lifespan.

## Conclusion

Eusocial insects and eusocial mammals share aging phenotypes despite phylogenetic divergence, but different mechanisms appear to have evolved to facilitate delayed aging in their nervous systems. The striking difference in worker and reproductive lifespan in eusocial insects is less pronounced in NMRs, further suggesting the evolution of multiple pathways to achieve long and healthy lifespans in eusocial taxa. To enable precise comparisons between vertebrate and invertebrate taxa, similar methodologies, such as quantifying levels of homologous biochemical markers of aging or comparisons among divergent taxa must be applied. Integration of theories of aging and development in eusocial and solitary species will enhance our understanding of how social organization shapes aging phenotypes and mechanisms that promote longevity.

## Author Contributions

YG, MM, and JT wrote the manuscript. YG and JT provided the funding. All authors contributed to the article and approved the submitted version.

## Conflict of Interest

The authors declare that the research was conducted in the absence of any commercial or financial relationships that could be construed as a potential conflict of interest.
